# Stable and reproducible MIP-ECL sensors for ultra-sensitive and accurate quantitative detection of Estrone

**DOI:** 10.3389/fbioe.2024.1329129

**Published:** 2024-02-09

**Authors:** Jie Cao, Xiao-Ying Chen

**Affiliations:** ^1^ Scientific Research and Experiment Center, Fujian Police College, Fuzhou, China; ^2^ Fuzhou University Postdoctoral Research Station of Chemistry, Fuzhou University, Fuzhou, China; ^3^ Fujian ShiMing Judicial Expertise Center, Fujian Police College, Fuzhou, China; ^4^ Regional Counter-Terrorism Research Centre, Fujian Police College, Fuzhou, China; ^5^ College of Environment and Safety Engineering, Fuzhou University, Fuzhou, China

**Keywords:** Estrone, molecular imprinting technique, electrochemical luminescence sensor, LC-MS/MS, environmental estrogen, human healthy risk assessment

## Abstract

Estrone (E1), as an endogenous estrogen, has a variety of physiological functions in human body and is of great significance to human health. On the other hand, it is a widely distributed and highly disturbing environmental endocrine disruptor in water. Therefore, there is an urgent need to develop a sensitive, rapid, and inexpensive method for the on-site determination of E1, which is not only for clinical diagnosis and treatment, but also for the investigation and monitoring of endogenous estrogen pollution in environmental water. In this study, Ru(bpy)_3_^2+^/MWCNTs/Nafion/gold electrodes were prepared by surface electrostatic adsorption and ion exchange. A molecularly imprinted membrane (MIP) with the capability to recognize E1 molecules was prepared by sol-gel method, and the electrodes were modified with MIP to form an electrochemical luminescence sensor (MIP-ECL). This method simultaneously possesses ECL’s advantage of high sensitivity and MIP’s advantage of high selectivity. Moreover, the addition of carboxylated multi-walled carbon nanotubes (MWCNT-COOH) improved the functionalization of the gold electrode surface and increased the binding sites of MIP. Meanwhile, the good conductivity of MWCNTs promoted electron transfer and further improved the sensitivity of the sensor. The sensor showed a wide linear interval in which the E1 concentrations can range from 0.1 μg/L to 200 μg/L, along with a high linear correlation coefficient (*R* ^2^ = 0.999). The linear regression equation of the sensor was Y = 243.64x-79.989, and the detection limit (LOD) was 0.0047 μg/L. To validate our sensor, actual samples were also measured by the reference method (LC-MS/MS), and it was found that the relative deviation of quantitative results of the two different methods was less than 4.1%. This indicates that the quantitative results obtained by this sensor are accurate and can be used for rapid *in situ* determination of E1 in clinical samples and environmental water.

## 1 Introduction

Estrone (E1) is a kind of female hormone secreted by the human body. It utilizes receptor-mediated pathways to bind to extracellular receptors in organisms. Upon binding to the promoter recognition site, it is transported to the nucleus to initiate the expression of target genes, thereby completing endocrine processes such as those involving estrogen and androgens. E1 serves as an important biomarker for pregnancy, women’s health before and after menopause, the early diagnosis of male prostate cancer, and female breast cancer, etc. (Salkho et al., 2018; Davis et al., 2019; Frederiksen et al., 2020; Mezzullo et al., 2020). E1 has stable chemical properties and is not easily degraded, thus it can exist and accumulate *in vivo* for a long time. Therefore, once in the environment, even microscale or trace concentrations of E1 still have irreversible physiological effects on biological reproduction and development, ultimately causing harm to human health. E1 is recognized as a highly active environmental endocrine disruptor (EDCs) consequently, there has been a rapid increase in studies on environmental monitoring and the biotoxicity mechanism of E1. It was reported that urban sewage, surface water, groundwater, receiving water, inland rivers, and estuaries had high levels of E1 pollution, which has adverse effects on the survival and reproduction of aquatic plants and animals (Ismail et al., 2019; Lei et al., 2020; Lu et al., 2020; Su et al., 2020). A different study demonstrated the presence of E1 in one of the four types of tissues analyzed in fish, namely, bile, liver, plasma, and muscle. In addition, studies have found E1 in smaller mussels (2–3 cm in length), which leads to increased human health risks due to their bioaccumulation in wild fish (Lv et al., 2019; Wolecki et al., 2019). It was also reported that the addition of E1 in feed could promote the productivity of livestock, reduce the mortality rate of animals, shorten the feeding cycle of animals, and promote the growth of animal products, which ultimately increased production. Despite this, the use of E1 and other estrogens as animal feed additives is strictly prohibited in all countries. To ensure the safety of livestock products and prevent contamination, food safety law enforcement agencies should employ advanced technical methods to strengthen the monitoring of illegal additives (Azzouz et al., 2011; Pu et al., 2019; Tomaž, 2019). In addition, it has been reported that E1 is commonly found in natural water sources. Therefore, monitoring of E1 in tap water is also necessary, and it is important to study the byproducts that are formed during the chlorine disinfection process used to treat drinking water (Borrull et al., 2020). In short, E1 is affecting all aspects of both the ecological environment and the general human health. Real-time analysis and accurate *in situ* quantification of E1 have become instrumental in E1-related medical diagnosis, toxicological research, and environmental regulation.

At present, the national standard detection method for E1 and other EDCs is high-performance liquid chromatography combined with tandem quadrupole mass spectrometry (LC-MS/MS). However, this method is associated with serious matrix interference and is not suitable for *in situ* or real-time dynamic detection. The required equipment is expensive, and the sample pretreatment process is complex, making the detection process cumbersome (Denver et al., 2019a; Denver et al., 2019b; Yuan et al., 2019; Nouri et al., 2020). These deficiencies in the standard detection method necessitate the innovation and development of a sensitive, high-specificity, and low-cost E1 detection method that works in real-time and *in situ* fashions.

Electrochemical luminescence (ECL) sensors are a simple and efficient detection method with high sensitivity. An ECL sensor is a new type of sensor based on electrochemically triggered light signal, combining electrochemistry and chemiluminescence technologies. By a certain voltage applied on the electrode, electrochemical reaction is used to directly or indirectly trigger chemiluminescence. This method collects light signals through instruments, such as photomultiplier tubes, and uses photoelectric conversion to measure the luminescence spectrum and intensity, thereby achieving qualitative and quantitative analysis toward an analyte. ECL sensors have a number of advantages: strong controllability, low cost, miniaturization of experimental devices, and *in situ* and real-time detection, which has become a greater priority in recent years (Liu H. B. et al., 2020; Liu et al., 2020b; Kamyabi and Moharramnezhad, 2020; Li et al., 2020). Traditional sensors based on antigen and antibody recognition often suffer from antibody inactivation and low antibody specificity, particularly for small molecules from those with weak auto-immunogenicity. To obtain antibodies for such molecules, it is necessary to prepare artificial haptens. Using artificial haptens to obtain antibodies can lead to decreased quality and immune cross-reactions, which can compromise the specificity of detection. As a result, preparing highly selective abiotic antibodies using molecular imprinting techniques (MIP) has become a hot topic. This approach is significant in enhancing the specificity of detection and improving the overall quality of the antibodies used in sensing (Liu et al., 2020c; He et al., 2020; Yuan et al., 2020; Zangiabadi and Zhao, 2020).

A novel MIP-ECL sensor is constructed by combining sensitive and controllable ECL sensor with highly specific MIP technology, which has a wide application prospect in life science, food safety, and environmental monitoring (Akgönüllü and Denizli, 2023; Akgönüllü et al., 2023; Liu and Ko, 2023; Ni et al., 2023). MIP-ECL sensors can be further divided into Ru-bpy system and Luminol-H_2_O_2_ system according to the different electrochemical luminescence signals. Ru-bpy has become one of the most commonly used ECL systems because of its good electrochemical reversibility and stability in both aqueous and non-aqueous phases, as well as its recyclability. MIP-ECL sensors can be divided into solid-state and non-solid-state luminescent electrode MIP-ECL sensors based on whether the luminescent reagent is immobilized on the electrode surface. In this work, Ru-bpy is immobilized on the electrode to prepare a solid electrochemical luminescence electrode, followed by incorporating a MIP film as a sensitive recognition element to achieve the integration of separation, enrichment, and detection of trace target analyte E1 in complex wastewater samples. This is a field with great research values and application prospects. However, MIP-ECL sensors have a relatively short development time, and many challenges are waiting for researchers to overcome, such as low current efficiency and weak detection signals.

The combination of MIP technology and ECL sensors had been developed in recent years. For example, Li et al. have constructed three MIP-ECL sensors for the specific detection of inorganic metal ions and organic small molecule compounds (Li S. H. et al., 2012; Li J. P. et al., 2012; Li et al., 2014; Li et al., 2015; Yang et al., 2016; Li et al., 2018). The LOD was 3.90 × 10^−13^ mol/L for Tb^3+^, 1.01 × 10^−12^ mol/L for Ni^2+^, and 2.35 × 10^−11^ mol/L for Be^2+^; and the LOD of clopyralid, gibberellin, and isoproturon was 3.7 × 10^−10^ mol/L, 3.45 × 10^−12^ mol/L, and 3.78 × 10^−12^ mol/L respectively. Zhang et al. developed MIP-ECL sensors (Wang et al., 2015; Wang et al., 2016; Zhang et al., 2017) with the LOD for fumonisin B1 and ochratoxin A were 0.35 pg/mL and 3.0 fg/mL, respectively. Jiang et al. developed an ECL sensor for the detection of ethylstilbestrol (DES) using a novel conjugated probe that integrated two recognition elements of magnetic molecularly imprinted polymers (MMIPs) and CdS quantum dots (CdS QDs) labeled with aptamers (Jiang et al., 2017). The LOD of DES by this new sensor is 0.1 pg/mL. Li et al. used a dual recognition system, which includes MIP and lincomycin aptamer, to construct a biosensor (Li et al., 2017) to obtain a LOD of 1.6 × 10^−13^ mol/L toward lincomycin. Wang et al. prepared a new environment-friendly MIP-ECL biosensor, which modified glassy carbon electrodes with nanomaterials, core-shell quantum dots (CdSeTe/ZnS), and MIP. Using this biosensor, trace levels of dopamine can be detected with a LOD of 3.3 × 10^−15^ mol/L (Wang et al., 2017). The specific performance parameters of the ECL sensor and MIP-ECL sensor cited above are summarized in Table 1. It can be seen that the linear ranges of MIP-ECL sensors and ECL sensors reported in various literature are mostly in the range of 10^2^ and 10^3^, and some can reach 10^4^ to 10^5^. The LOD values were mostly at 1 ng/mL or 1 pg/mL and could reach 1 fg/mL. The new E1 MIPs-ECL sensor developed in this work is expected to make its linear range and LOD value comparable to other similar sensors through optimizing various measurement conditions. It is further intended that the sensor has excellent performances, such as high specific recognition of E1 in complex wastewater samples, and high stability, accuracy, and reproducibility of quantitative results.

**TABLE 1:** Summary of performance parameters of ECL sensor and MIP-ECL sensors.

Number	Sensor type	Linear range	LOD value	Target analyte	Actual sample type	References
1	MIPs-ECL Sensor	8×10^−13^ mol/L to 4 × 10^−9^ mol/L	3.90 × 10^−13^ mol/L	Tb^3+^	sea water samples	Li et al. (2018)
2	MIPs-ECL Sensor	3 × 10^−12^ mol/L to 6.0 × 10^−9^ mol/L	1.01 × 10^−12^ mol/L	Ni^2+^	food samples, including apples, carrots and grapes	Yang et al. (2016)
3	MIPs-ECL Sensor	7 × 10^−11^ mol/L to 8 × 10^−9^ mol/L	2.35 × 10^−11^ mol/L	beryllium	real water samples	Li et al. (2015)
4	MIPs-ECL Sensor	1 × 10^−9^ mol/L to 8 × 10^−7^ mol/L	3.7 × 10^−10^ mol/L	Clopyralid	vegetables	Li et al. (2014)
5	MIPs-ECL Sensor	9 × 10^−11^ mol/L to 5.1 × 10^−9^ mol/L	3.78 × 10^−12^ mol/L	Isoproturon	Water samples	Li et al. (2012a)
6	MIPs-ECL Sensor	1 × 10^−11^ mol/L to 3 × 10^−9^ mol/L	3.45 × 10^−12^ mol/L	Gibberellin A3	Beer samples	Li et al. (2012b)
7	MIPs-ECL Sensor	0.001 ng/mL to 100 ng/mL	0.35 pg/mL	fumonisin B1	milk and maize samples	Zhang et al. (2017)
8	MIPs-ECL Sensor	1 × 10^−5^ ng/mL to 1.13 ng/mL	3.0 fg/mL	Ochratoxin A	real samples of corn and human serum	Wang et al. (2016)
9	MIPs-ECL Sensor	0.1 ng/mL to 10 ng/mL	0.03 ng/mL	Ochratoxin A	Corn samples	Wang et al. (2015)
10	MIPs-ECL Sensor	0.3 pg/mL to 1 × 10^−5^ pg/mL	0.1 pg/mL	diethylstilbestrol	complex foodstuff matrix	Jiang et al. (2017)
11	MIPs-ECL Sensor	5 × 10^−12^ mol/L to 1 × 10^−9^ mol/L	1.6 × 10^−13^ mol/L	lincomycin	meat samples	Li et al. (2017)
12	MIPs-ECL Sensor	1 × 10^−14^ mol/L to 2.5 × 10^−12^ mol/L	3.3 × 10^−15^ mol/L	Dopamine	human serum	Wang et al. (2017)
13	ECL Sensor	3 × 10^−14^ mol/L to 8 × 10^−8^ mol/L	4.4 × 10^−15^ mol/L	Imidacloprid	different water (such as Tap water, Well water, River water) and fruit samples (such as Apple, cucumber, tomato)	Kamyabi and Moharramnezhad (2020)
14	ECL Sensor	0.001 μmol/L to 1.0 μmol/L	0.5 nmol/L	glyphosate	water samples, including local river water and lake water	Liu et al. (2020a)
15	ECL Sensor	0.01 ng/mL to 100 ng/mL	3.1 pg/mL	chloramphenicol	honey and shrimp sample	Li et al. (2020)
16	ECL Sensor	0.5 pmol/L to 1 nmol/L	0.17 pmol/L	hepatitis C virus (HCV) gene	human serum	Liu et al. (2020b)

The research on MIP-ECL sensors is primarily in early stage of exploring new detection methods in the laboratory. Studies on the sophisticated development of the sensors, thorough investigation on the sensor’s performance, and the validation of quantitative results with the standard LC-MS/MS detection method are still missing. As far as we know, the development and use of MIP-ECL sensors for specific qualitative and quantitative analysis of E1 have not been researched yet.

The study utilized a sol-gel method to prepare a MIP electrode-modified layer and added carbon nanotubes to enhance the detection signal. This approach helps to overcome the limitations of traditional biosensors that rely heavily on the quality of antibodies for the specific detection. The use of MIP-ECL sensors negates the potential impact that the specificity and stability of antibodies, ion strength, pH value, and the presence of interfering substances in the detection medium could have made on the analysis results. Moreover, the modified layer of the MIP electrode has the advantages of a stable coating, acid and alkali resistance, superior repeatability and reproducibility, and reusability post-recovery.

## 2 Materials and methods

### 2.1 Chemicals and reagents

Estrone (E1), methyl alcohol, Tetraethylorthosilicate (TEOS), methyltrimethoxysilane (MTMOS), phenyltrimethoxysilane (PTMOS), Nafion (a solution of 5% lower fatty alcohols and water), multiwalled carbon nanotube (MWCNT), estradiol (E2), bisphenol A (BPA), Nonylphenol (NP), and bis-terpyridyl ruthenium complex powder (Ru(bpy)_3_Cl_2_^.^6H_2_O, denoted as Ru-bpy) were obtained from Sigma-Aldrich (St. Louis, MO, United States). Dimethylsulfoxide, dipotassium phosphate (K_2_HPO_4_), monopotassium phosphate (KH_2_PO_4_), phosphoric acid, sodium hydroxide (NaOH), tripropylamine (TPA), concentrated sulfuric acid, concentrated nitric acid, hydrochloric acid (HCl) and absolute ethyl alcohol were purchased from Sinopharm Chemical Reagent Co., Ltd. (Shanghai, China). E1 stock solution was prepared with methyl alcohol and stored at 4°C. Carboxylated MWCNTs (MWCNT-COOH) were prepared according to reference (Ding et al., 2013), and the specific process was referred to the text in Section 2.4. MWCNT-COOH solution was prepared using dimethylsulfoxide. Phosphate-buffered solution (PBS, 200 mL, pH 7.4, 0.1 mol/L) was made from 1.36 g KH_2_PO_4_ and 79 mL NaOH (0.1 mol/L) with ultrapure water. The solution of Ru-bpy was prepared by dissolving bis-terpyridyl ruthenium complex powder in ultrapure water (see description below).

### 2.2 Apparatus

In this study, the CHI660E electrochemical workstation produced by CH Instruments, Inc. (Shanghai, China) was used for electrochemical measurements. The signal of ECL was detected by a BPCL ultra-weak luminescence analyzer (Beijing, China) developed by the Institute of Biophysics, Chinese Academy of Sciences. A three-electrode system was used for ECL detection, in which Ag/AgCl (saturated KCl solution) was used as a reference electrode; a platinum wire was used as a counter electrode; and MWCNT-COOH modified gold electrode was used as a working electrode. Atomic Force Microscope (AFM) was operated with an Agilent 5,500 multifunctional scanning probe microscope (Foster City, CA, United States). Ultrapure water is the tap water that has been purified through the Aquapro Water purifier (Chongqing, China) and subsequently the Milli-Q IQ 7000 Laboratory Water System (Massachusetts, United States). A Shimadzu L20A high-performance liquid chromatography (Kyoto, Japan) combined with AB SCIEX 3200QTRAP tandem quadrupole Mass Spectrometer (Framingham, MA, United States) was used for quantitative analysis of E1 in samples, and the results were compared and used to validate the new MIPs-ECL sensor method.

### 2.3 Synthesis of MIPs

The MIP precursor solution, namely, non-molecularly imprinted polymers (NIPs), were prepared as the following. TEOS, MTMOS, PTMOS, anhydrous ethanol, 0.1 M HCl, and ultrapure water were added in sequence to a 5 mL centrifuge tube. The corresponding volumes were 200 μL, 60 μL, 60 μL, 200 μL, 10 μL (0.1 mol/L), and 200 μL, respectively. Thus, the optimal volume ratio of precursor solution, in terms of TEOS, MTMOS, PTMOS, CH_3_CH_2_OH, 0.1 M HCl, and H_2_O, is 20:6:6:20:1:20. The solution was then mixing by ultrasonic dispersion for 1 h at 25°C to obtain a uniform and transparent sol (NIPs). MIPs were prepared by mixing NIPs (400 μL) with a 100 mg/L E1 solution of 40 μL, followed by the same ultrasonic process previously described. The optimal volume ratio of each component in the MIP precursor solution to template molecules E1 is: TEOS:MTMOS:PTMOS:CH_3_CH_2_OH: HCl:H_2_O: E1 = 20:6:6:20:1:20:10.

### 2.4 Preparation of carboxylated MWCNTs

Prepare a mixture of 1.2 mL concentrated nitric acid and sulfuric acid (1:3 v/v) and 10 mg MWCNTs. After heated refluxing at 90°C for 4 h, the product was repeatedly washed to neutral pH with pure water, filtered with 0.25 µm fiber filter paper, and dried using a vacuum drying oven at 40°C. The resulting carboxylated MWCNTs was grounded into powder for storage under an infrared lamp. The acid-treated MWCNTs (1.0 mg) was added to 1 mL dimethyl sulfoxide, and a homogeneous and stable suspension was obtained after ultrasonic dispersion for 20 min.

### 2.5 Preparation of MIP-ECL sensor

**FIGURE 1:**
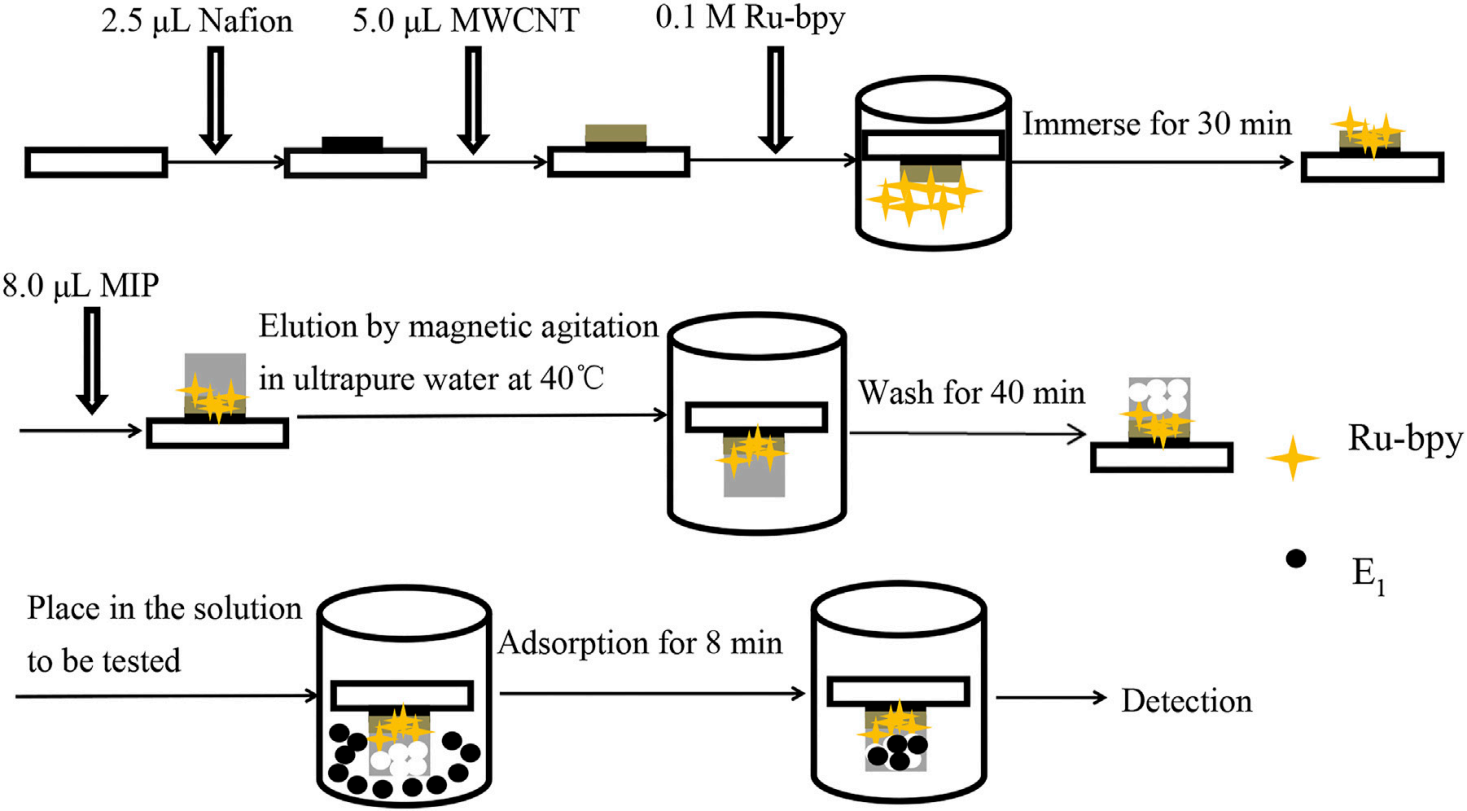
The electrode modification and detection schematic diagram of MIP-ECL sensor.

**FIGURE 2:**
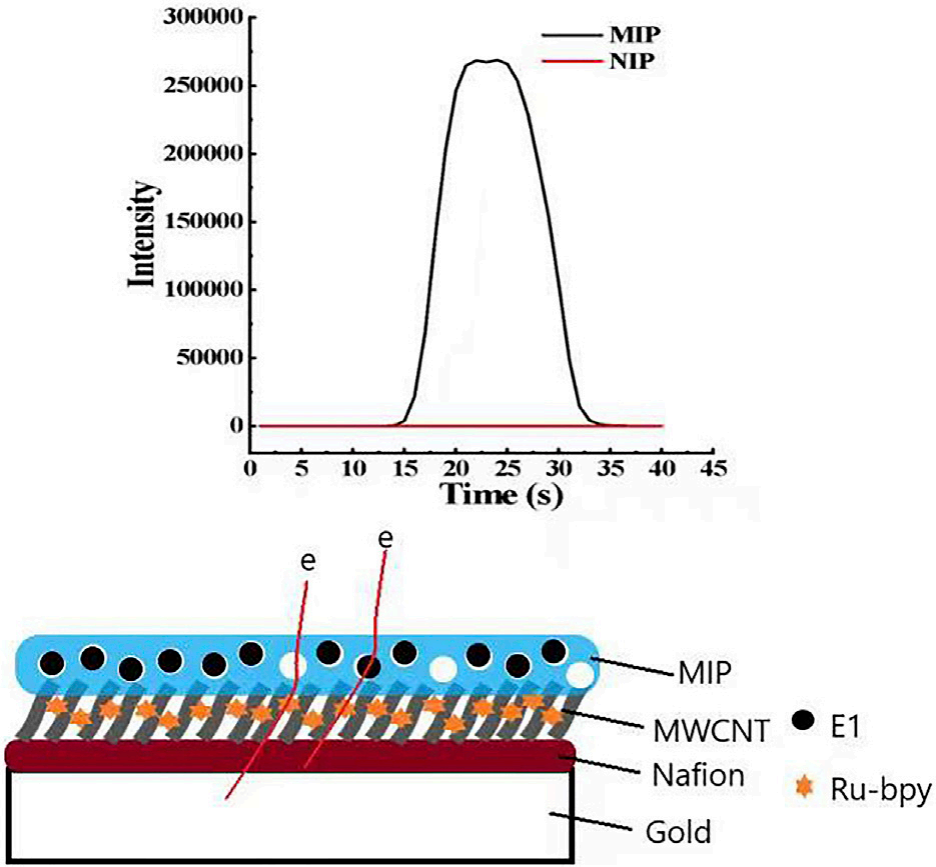
The detection principle diagram of MIP-ECL sensor.

The schematic diagram of the electrode modification and detection principle of the MIP-ECL sensor are shown in Figures 1, 2.

Prior to electrode modification, the gold electrode was polished to a specular surface using 0.3 μm Al_2_O_3_ sandpaper followed by 0.05 μm Al_2_O_3_ sandpaper. The electrode then underwent two rounds of ultrasonic cleaning with anhydrous ethanol and ultrapure water for 15 min. The electrode was then dried with filter paper.

The electrode modification process of MIP-ECL sensor is divided into two steps. The first step is to immobilize the ECL complex on the surface of the gold electrode to obtain an ECL solid-phase electrode. The second step is to deposit the MIP membrane on an ECL solid-phase electrode to prepare an ECL-MIP sensor.

As shown in Figure 1, Nafion solution (2.5 µL) was coated to the surface of the electrode and dried at 25°C, followed by depositing 5 µL MWCNT-COOH solution and being dry at 25°C. Next, the electrodes were placed in Ru-bpy solution for 30 min and then removed for being rinsed and dried at 25°C. The first step of the preparation of Ru-bpy/MWCNT/Au ECL solid-state electrode was completed. Then, the Ru-bpy/MWCNT/Au electrode was coated with NIPs or MIPs (8 µL), and then dried at 25°C. Finally, the coated NIPs/Ru-bpy/MWCNT/Au electrode or MIPs/Ru-bpy/MWCNT/Au electrode was cleaned in 40°C ultrapure water with magnetic stirring for 40 min to remove template molecules (E1) from the imprinted hole as well as adsorbed impurities. The second step was completed, in which MIP-ECL sensors are with three-dimensional cavities that can recognize E1 molecules. On contrast, the control NIP-ECL sensors with almost no cavities are obtained.

The detection principle is shown in Figure 2, in which the schematic diagram of the electrode surface with a layer-by-layer modification can be clearly seen. The bottom layer is the gold electrode, followed by Nafion layer, MWCNT-COOH layer, and MIP or NIP layer. Since the MIP layer has holes, it is suggested that electron transfer was facilitated through holes that were occupied by E1 molecules to yield a high signal. Negligible signal was observed for the NIP-ECL sensor, likely due to the absence of electron transfer routes (through the holes and the bound E1 molecules).

### 2.6 ECL measurement

Before sample testing, the MIP-ECL sensor was thoroughly cleaned with ultrapure water and immersed in a sample solution. The sensor was reacting with the E1 solution for 8 min, then removed and rinsed off the non-specific bound E1. In measurement, it was placed into a PBS/TPA buffers solution (without E1), in which 10 µL of TPA was added in to 3 mL PBS, to test the ECL signal response. The continuous potential scanning range of cyclic voltammetry (CV) was −1.2–2.0 V. The current range of the Ultra-Weak chemiluminescence analyzer is −1,000∼-900 V. All experiments were performed at a temperature of approximately 25°C in the laboratory.

### 2.7 LC-MS/MS determination

**FIGURE 3:**
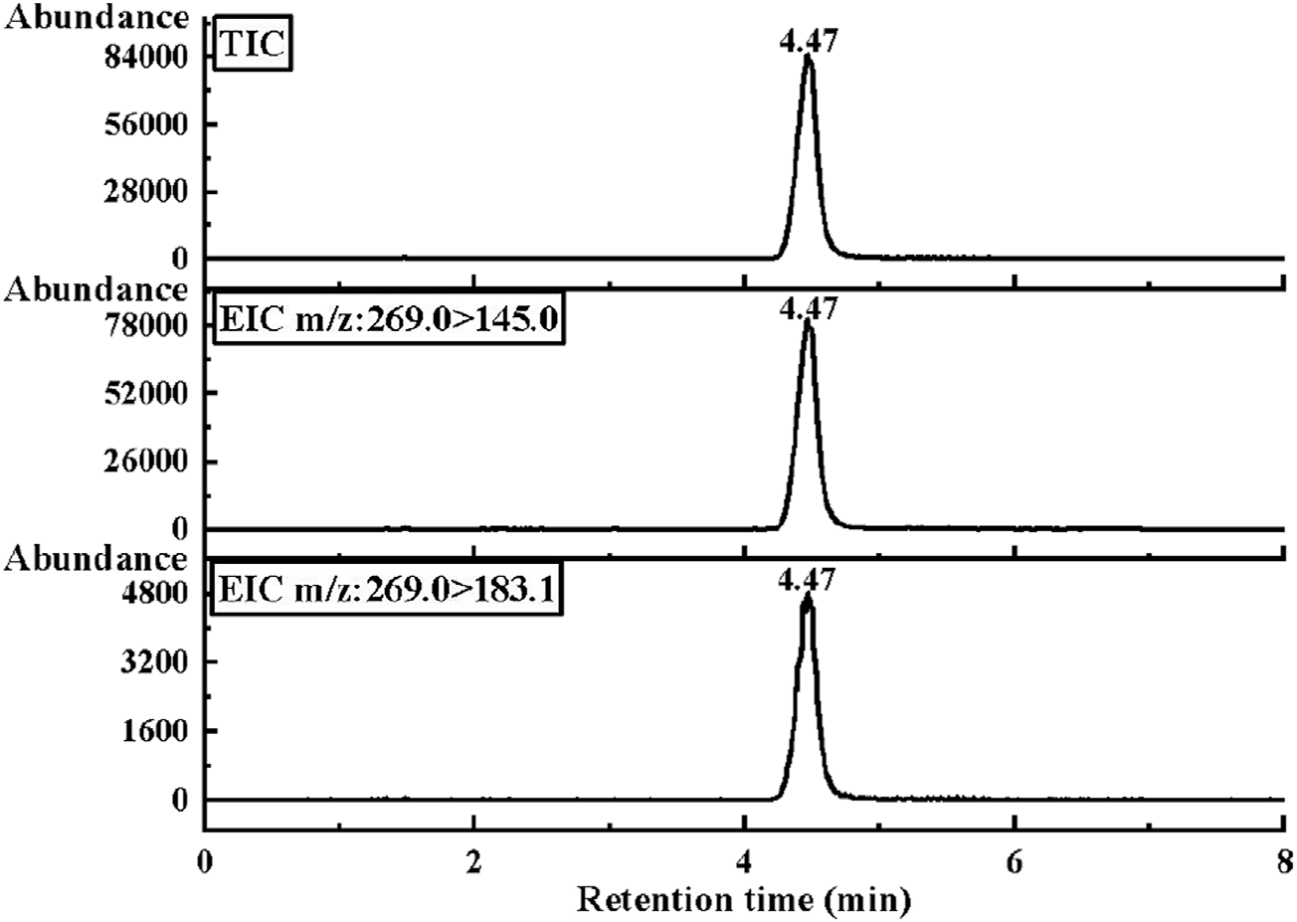
The MRM mode of LC-MS/MS was used to collect the optimal chromatograms of target molecule E1, including the chromatograms of two characteristic ion pairs and TIC chromatograms.

Under the optimized conditions in Table 2, the chromatograms of the total ion current (TIC) and two characteristic ion pairs of 0.2 μg/mL E1 are determined by the LC-MS/MS method, as shown in Figure 3. The retention time of E1 is shown as 4.47 min.

**TABLE 2:** Optimal conditions for detecting E1 by LC-MS/MS.

	Method	Solid phase microextraction		
Sample pretreatment	Environmental water sample	filtered by 0.25 µm filter membrane		
	Extraction column type	C18		
	Column activated solvent	successively with 5.0 mL methanol and 10.0 mL ultrapure water		
	Sample volume	After the activation extraction column, water sample (50 mL) or spiked water sample (10 mL) was flowed		
	Column cleaner	successively with 5.0 mL ultrapure water and 5.0 mL n-hexane		
	Column elution solvent	5.0 mL dichloromethane		
	Sample injection	Step 1: The elution solution containing E1 is slowly blown dry in nitrogen at 40°C. Step 2: 1.0 mL methanol was added to redissolve the attachment. Step 3: The sample was taken to a 1.5 mL sampling bottle for LC-MS/MS analysis		
HPLC condition	Column	Phenomenex: 5 μm, 4.6 mmi.d.×150 mm		
	Mobile phase	The mobile phase double channels include channel A for ultra-pure water and channel B for methanol		
	Gradient elution	initial condition 10% B, 0–1 min 40% B, 1–3 min 80% B, 3–9 min 40% B, and 9–10 min 10% B		
	Flow rate	0.3 mL/min		
	Oven temperature	30°C		
MS/MS condition	Analytical mode	MRM		
	E1 characteristic ion pair	DP	CE	CXP
	269/145	−61.26 V	−47.45 V	−2.31 V
	269/183.1	−83.44 V	−47.53 V	−2.40 V

## 3 Results and discussion

### 3.1 Characterization of MIPs layer

**FIGURE 4:**
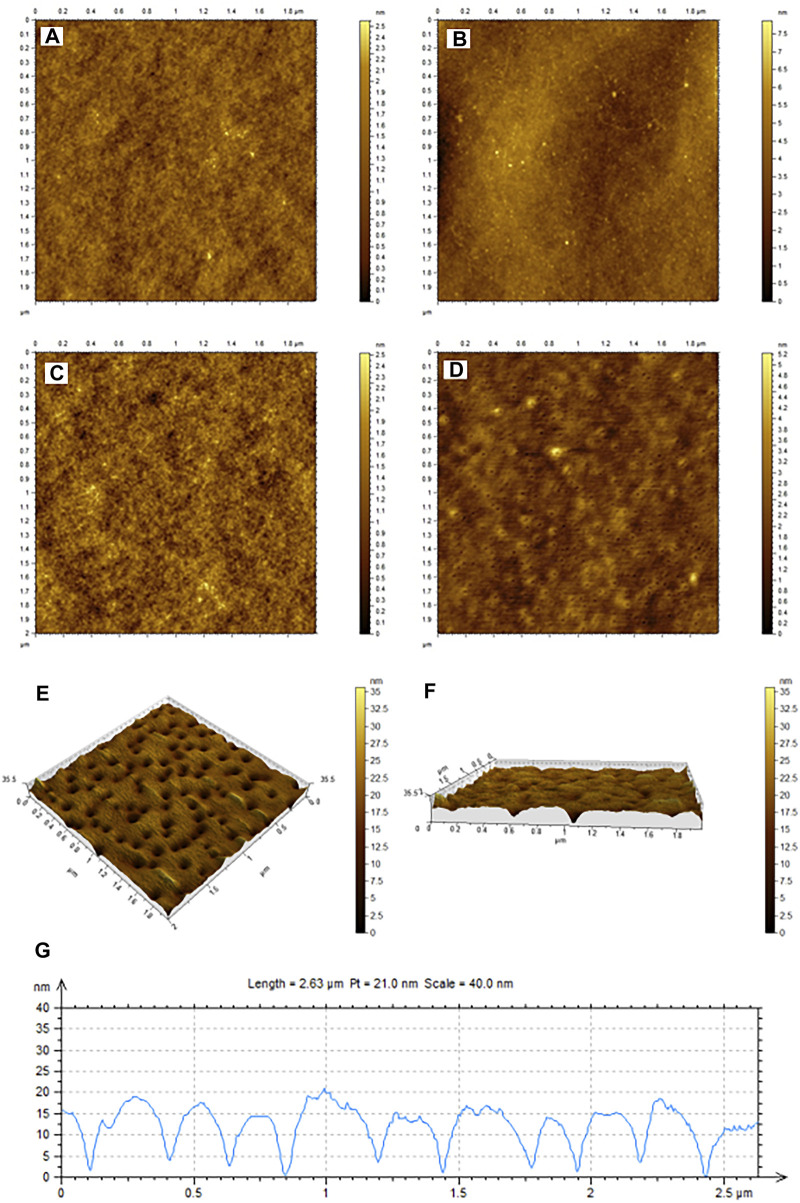
AFM characterization of NIPs **(A,B)** MIPs **(C,D)** before **(A,C)** and after **(B,D)** rinsing. **(E)** Is the three-dimensional diagram of MIP holes left by the elution of template molecule E1. **(F)** Shows the cross-section of the MIPs layer in **(E)**. **(G)** Shows the width and depth of 10 holes, which were randomly selected in **(E)**.

The glass slides modified by NIPs or MIPs were observed with AFM, as shown in Figure 4. As seen in Figures 4A, C, both NIP and MIP layers appear to be of rigid structure and smooth surface. After elution, no significant change was observed for the NIP layer (Figure 4B), whereas a mesoporous surface was observed for the MIP layer (Figure 4D), indicating that the template molecule E1 in MIPs was successfully removed after elution in ultrapure water at 40°C. The removal of the template molecules leaves holes and recognition sites in the rigid network structure of the polymer.

Furthermore, the three-dimensional view of MIP holes left by the elution of template molecule E1 was shown in Figure 4E. Figure 4F shows the cross-section of the MIPs layer in Figure 4E. Figure 4G shows the width and depth of 10 holes, which were randomly selected from Figure 4E. According to Figure 4G, the average cavity depth and width of the imprinted material after elution are 12.3 nm and 100 nm respectively, which match the spatial structure of the template molecules.

The sensor uses a gold electrode, and four layers are modified on the surface of a gold electrode. The first layer was formed by coating the electrode with a 2.5 µL Nafion solution, which serves as the crosslinking agent. A 5 µL of MWCNT-COOH suspension solution was then deposited onto the electrode. The resulting modified electrode was immersed in a Ru-bpy solution for 30 min, and then washed and dried. The porous structure of MWCNT was expected to adsorb Ru-bpy luminescent reagent, and the luminescent reagent was immobilized on the electrode surface. The luminescent reagent Ru-bpy is a small molecule which should be adsorbed inside the holes structure of MWCNT, so the thickness of Ru-bpy is not taken into consideration. The fourth layer is formed by coating a 8 µL MIP or NIP suspension. The thickness of the fourth layer can be inferred as c.a. 40 nm based on Figures 4F, G.

The specific recognition mechanism is as follows. Firstly, the MIP layer has the function of specific recognition, separation, and enrichment of the target analyte molecule E1. Bound E1 is more likely to contact with the luminescent reagent Ru-bpy in the MWCNT hole of the electrode because of the proximity. The relationship between the change in luminescence intensity, generated by the oxidation-reduction reaction under electrocatalysis, and the concentration of the target E1 is the basis for E1 quantification. Secondly, according to the molecular structure, E1 contains one benzene ring, two C6 rings, one C5 ring, and one carbonyl group. The molecular structure is stable, and the middle ring structure is hydrophobic. In this study, the sol-gel method was used to prepare MIP functional films. The functional monomers used in this method were PTMOS and MTMOS. Due to the existence of benzene ring on the template molecule E1, a π-π conjugated effect with hydrophobic PTMOS could occur. MTMOS can improve the stability and hydrophobicity of sol-gel and make E1 with circular hydrophobic structure in the middle of the molecule more easily enter the MIP hydrophobic holes (Pichon and Chapuis-Hugon, 2008). In summary, the process of MIP layer recognition of target E1 in this study includes the minimum spatial resistance between MIP holes space structure and the target E1, the electrostatic attraction generated by the π-π conjugation effect between the functional monomer PTMOS and the E1 benzene ring, and the hydrophobic force generated by the E1 molecule and the functional monomer MTMOS. Driven by the joint action, the recognition of the target E1 is easy, stable, and efficient.

The prepared MIP functional film has both thermal and chemical stability. Firstly, compared to biological receptors or antibodies, MIPs have higher chemical and physical stability; are easier to obtain; and have lower costs. Secondly, the sol-gel technology is combined with MIPs to prepare sol-gel MIP functional film in this study. The MIP film prepared by this method has a high degree of crosslinking compared to MIPs films prepared by organic polymerization and other methods. It can form a strong inorganic three-dimensional network structure, which is stable and resistant to high temperature and chemical reagents, such as acid, alkali, and salt. Thirdly, it can be found from the formation mechanism that the properties of sol-gel MIP materials are stable. The specific formation steps include: 1) The functional monomers were mixed uniformly in the liquid phase, and underwent chemical reactions, such as hydrolysis and condensation. A stable transparent sol system in the solution was formed thereafter. 2) The gel was slowly polymerized between aged colloidal particles to form a gel with three-dimensional network structure. The gel was dried or sintered to prepare molecular and even nanostructured materials (Marx and Liron, 2001; Zhang et al., 2005). In addition, the functional monomers and crosslinkers should be selected according to the structural characteristics of the template molecules during the preparation of the sol-gel MIP functional film. For example, consideration should be given to the following factors: whether template molecules have benzene rings or conjugated structures, and whether template molecules can generate π-π conjugation effects with hydrophobic functional monomers. This is to improve the stability and hydrophobicity of MIPs. According to the above principles, it can improve the specific selectivity of the MIP film to the target molecule, but also the stability of the cross-linked MIP film.

### 3.2 ECL characteristics of new E1 sensor

**FIGURE 5:**
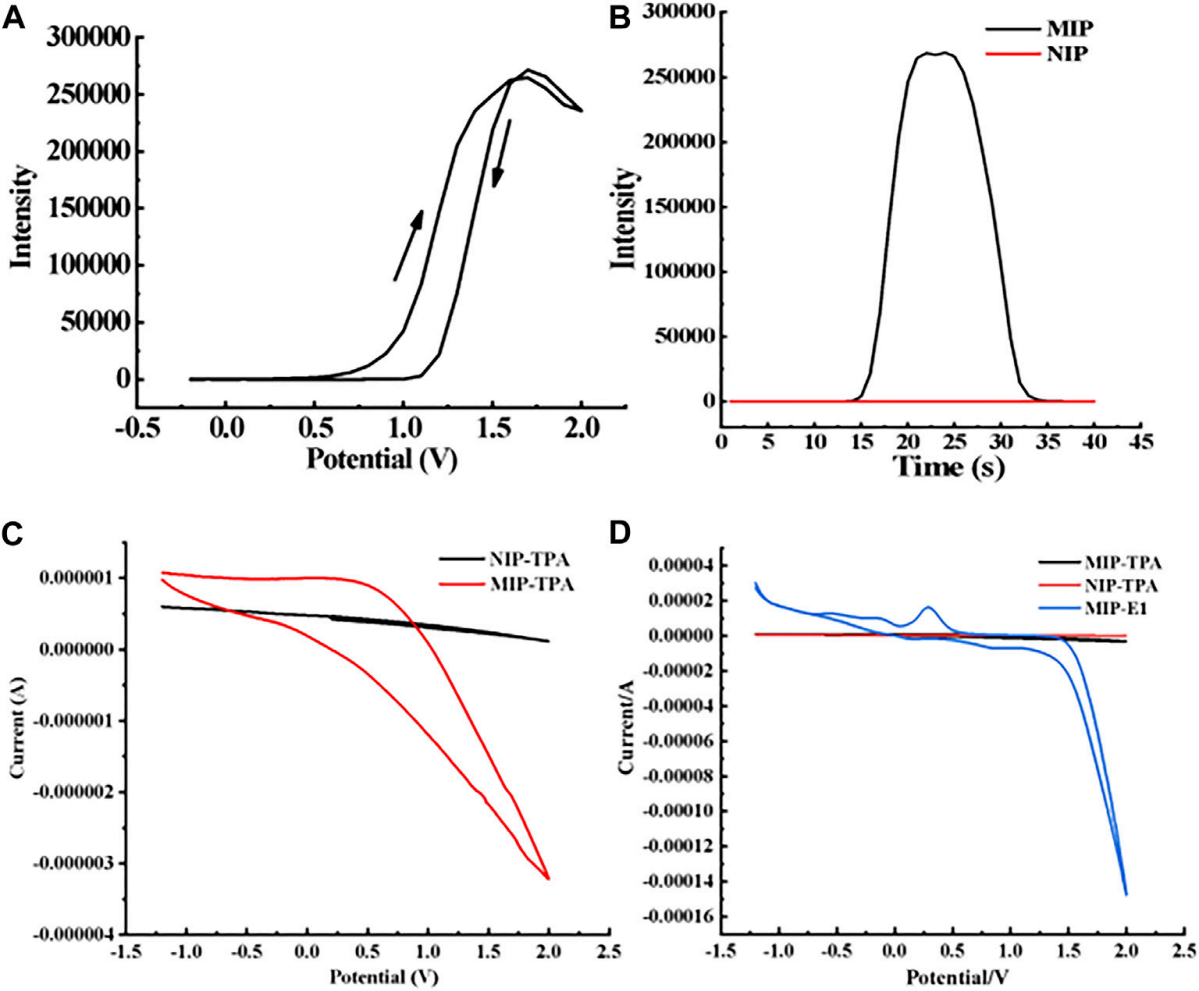
ECL characterization of MIP- and NIP-modified electrodes. **(A)** Relationship between ECL intensity of MIP-modified electrode and scanning potential when the concentration of target molecule E1 is 1 mg/L **(B)** ECL intensity of MIPs- and NIPs-modified electrodes when the concentration of target molecule E1 is 1 mg/L. **(C)** In the absence of E1, the corresponding current diagram of TPA when the NIP/Ru-bpy/MWCNT/Au electrode and MIPs/Ru-bpy/MWCNT/Au electrode are used. **(D)** The corresponding current diagram of E1 and TPA when the NIP/Ru-bpy/MWCNT/Au electrode and MIPs/Ru-bpy/MWCNT/Au electrode are used.

As shown in Figure 5A, the luminescence intensity varies with voltage when the MIP-ECL sensor is used to detect 1 mg/L E1 solution, and the luminescence signal begins to appear at a voltage of 1.0 V, which is consistent with that in other reports (Wang et al., 2015; Wang et al., 2016; Zhang et al., 2017). Subsequently, the luminescence intensity increases rapidly, reaching its maximum at about 1.52 V. The results show that the optimal redox potential for our measurements is about 1.52 V.

When the new sensor is used to detect 1 mg/L E1 solution—if its electrode is modified with NIP—the ECL signal is almost zero, as shown in the red curve in Figure 5B. If its electrode is modified with MIP, the ECL signal is significantly increased, as shown in the black curve in Figure 5B. We hypothesized that the enhancement in ECL signal was attributed to a facile electron transfer facilitated by the holes and E1 molecules within the MIP films. Furthermore, the imprinting factor (IF) of electrode modified MIP films can be calculated. IF is defined as the binding ratio of MIPs to NIPs, and the calculation formula is as follows.

IF = K_MIP_/K_NIP_ (K_MIP_ and K_NIP_ represent the binding capacities in a monolayer polymer surface).

According to the above definition and calculation formula of IF, along with the experimental results of our study, we calculated IF of the electrode-modified E1-MIP film. IF(E1) is equal to the ratio between the average ECL signal intensity generated by the electrode modified E1-MIP film and E1-NIP film, which is about 268,897 to 60. The final calculated IF(E1) of E1-MIP film is 4481.62.

To verify our hypothesis, we investigated the electron transfer activities on the modified electrodes by electrochemical CV. The signal indicator, Ru-bpy, was either placed in solution or immobilized on the electrode surface. No interference to the electron transfer on MWCNT/Nafion modified electrode, as we compared its electrochemical response (Supplementary Figure S1A-b) to that on the bare gold electrode (Supplementary Figure S1A-a). As expected, the immobilized Ru-bpy on MWCNT/Nafion/Au electrode showed a slight lower reduction potential than that in solution, seen Supplementary Figure S1B.

TPA has been proved to be able to electrochemically amplify/catalyze Ru-bpy’s reduction signal (see details in the following Section 3.3.1), so that it was employed to assess the electron transfer through the NIP or MIP film. While the Ru-bpy was “sandwiched” between the NIP (or the MIP) and MWCNT/Nafion layers, TPA was placed in the electrolyte. It was found that the electrochemical signal was significantly enhanced when MIP/Ru-bpy/MWCNT/Au electrode is used (Figure 5C), in comparison to the negligible signal on NIPs/Ru-bpy/MWCNT/Au electrode. This indicates that the MIP layer facilitates the electron transfer likely due to the porous structure.

Interestingly, the reduction peak current of Ru-bpy increases after adding E1, which indicates that E1 has an electrocatalytic effect on the reduction current of Ru-bpy (Supplementary Figure S1C). This phenomenon was consistent with a relevant report by Kuzikov et al. (2020).

The current signals were detected with MIPs/Ru-bpy/MWCNT/Au electrodes in the present of E1 and TPA respectively. The electrocatalytic current signal by E1 was found about 15 times higher than that of TPA, see Figure 5D. Taken together, the enhanced ECL signal in Figure 5B was likely attributed to the electrocatalysis by E1 molecules but also the facilitated electron transfer by the porous MIP film to allow TPA access and react with the immobilized Ru-bpy.

### 3.3 Optimization of E1 sensor test system

#### 3.3.1 Optimization of co-reagents in solution

**FIGURE 6:**
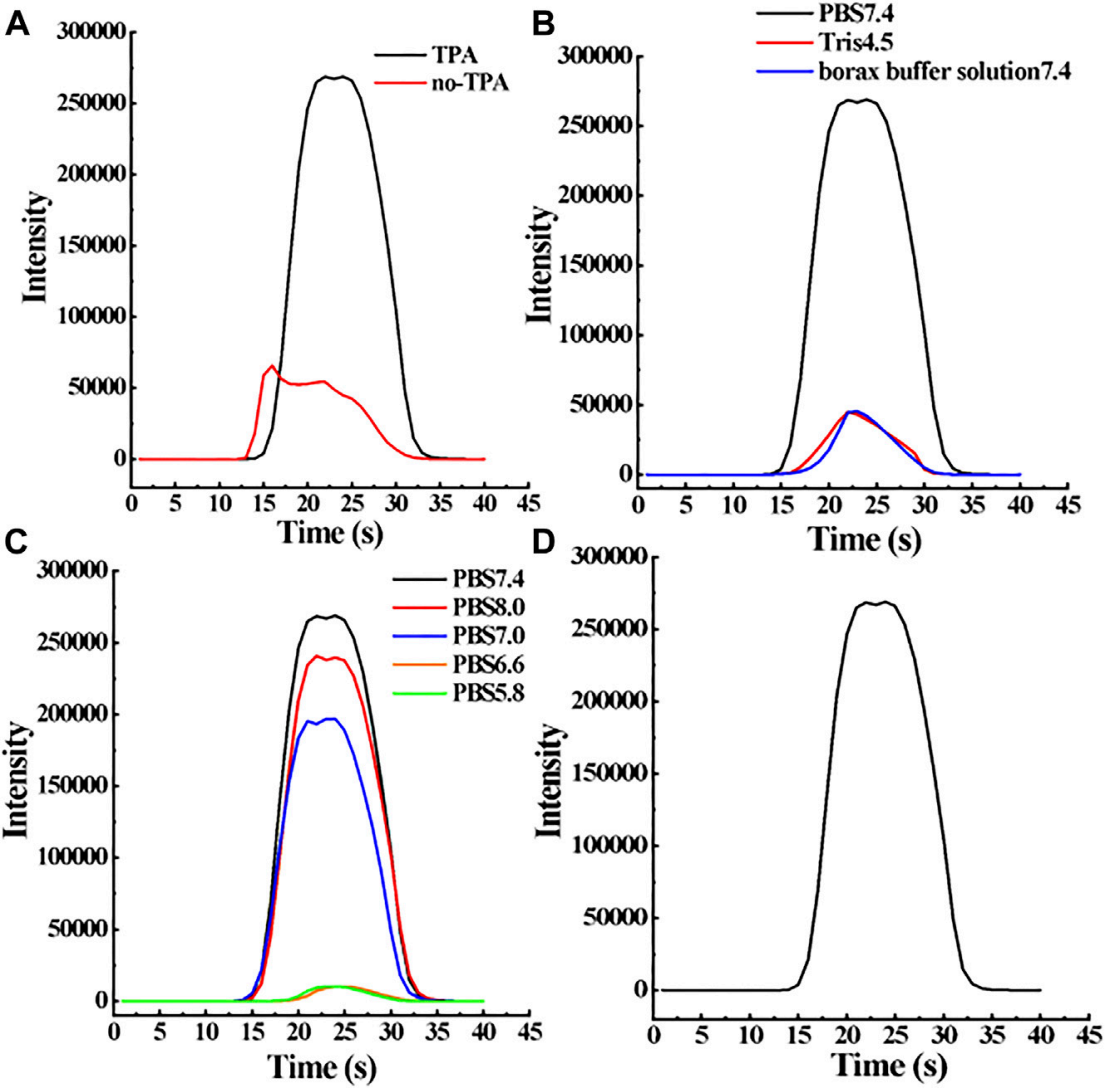
Optimization of key parameters in the new E1 sensor analysis system. Specific parameters include co-reactant TPA **(A)**, buffer solution **(B)**, and pH **(C)**. When the concentration of the target molecule E1 is 1 mg/L, the change of these parameters will affect the ECL response intensity of the sensor, namely, the sensor sensitivity. **(D)** ECL diagram of 1 mg/L E1 under optimal experimental conditions.

As shown in Figure 6A, the luminescence intensity of 1 mg/L E1 solution is 5 times that of the solution without TPA. TPA can be oxidized to produce the highly active cation TPA(+). Then TPA(+) is rapidly decomposed into TPA free radicals to react with the oxidized Ru-bpy(3+) to yield the excited Ru-bpy(2+)*, which emits light at about 620 nm and converts back into Ru-bpy(2+). The reaction re-occurs and the signal of Ru-bpy is therefore enhanced.

Taking a closer examination of the electrochemical reaction when both TPA and E1 are in presence. We speculate that the reaction mechanism is as follows. First, TPA can be oxidized to generate the highly active cation TPA(+) and TPA(+) can be rapidly decomposed into TPA free radicals. Second, E1 can reduce Ru-bpy(2+) to Ru-bpy(3+). Thirdly, Ru-bpy(3+) can react with TPA radicals to regenerate excited Ru-bpy(2+)* ions, which results in the enhancement of the ECL signal.

The inclusion of TPA as a co-reactive agent in the E1 solution for ECL resulted in a substantial increase in the ECL response signal. Furthermore, the MIP-ECL sensor showcased exceptional selectivity towards E1 molecules, thereby underscoring its utility as a reliable detection tool.

#### 3.3.2 Optimization of solution medium

An electrolyte medium is a key factor in the electrochemical analysis, which can affect the redox rate of the target compound on the electrode and the transfer rate of the target compound in the electrolytic cell. Therefore, it is very important to select the appropriate electrolyte to improve the sensitivity of the ECL sensor. In order to select the best medium conditions for E1 measurement, PBS (pH 7.4), Tris (pH 4.5), and borax buffer solution (pH 7.4) were used as a medium to prepare 1 mg/L E1 solution for the detection of MIP-ECL sensor. According to the results in Figure 6B, the ECL signal of 1 mg/L E1 solution prepared with PBS (pH 7.4) was the strongest. Therefore, PBS solution is used as the ECL detection solution in this experiment.

#### 3.3.3 Optimization of pH value of the solution to be tested in sensor system

E1 solutions with the same concentrations were prepared by PBS with different pH values, the luminescence intensity was then studied through MIP-ECL sensors to select the optimal pH value of PBS solutions. In this study, 0.1 mol/L PBS with pH values of 5.8, 6.6, 7.0, 7.4, and 8.0 were used to prepare 1 mg/L E1 solution. As shown in Figure 6C, when the pH range of the buffer solution is 7.0–8.0, the luminescent signal of E1 detected by the MIP-ECL sensor is relatively strong, however, when the pH range is 5.8–6.6, the luminescent signal of E1 detected by MIP-ECL sensor is relatively weak. Therefore, the optimal solution pH value of E1 for detection through the MIP-ECL sensor is neutral or slightly alkaline. In this experiment, a pH of 7.4 was selected as the detecting condition.

After the optimization experiments of co-reagent, buffer solution, and pH the following was determined: TPA as a co-reagent can greatly improve the luminescence intensity of the MIP-ECL sensor for E1. The luminescence signal is strongest when the pH value of PBS is 7.4. Sensor test systems are all under the above optimal conditions. When 1 mg/L E1 solution is detected, ECL response signal curve is shown in Figure 6D.

### 3.4 Establishment of quantitative linear regression equation

**FIGURE 7:**
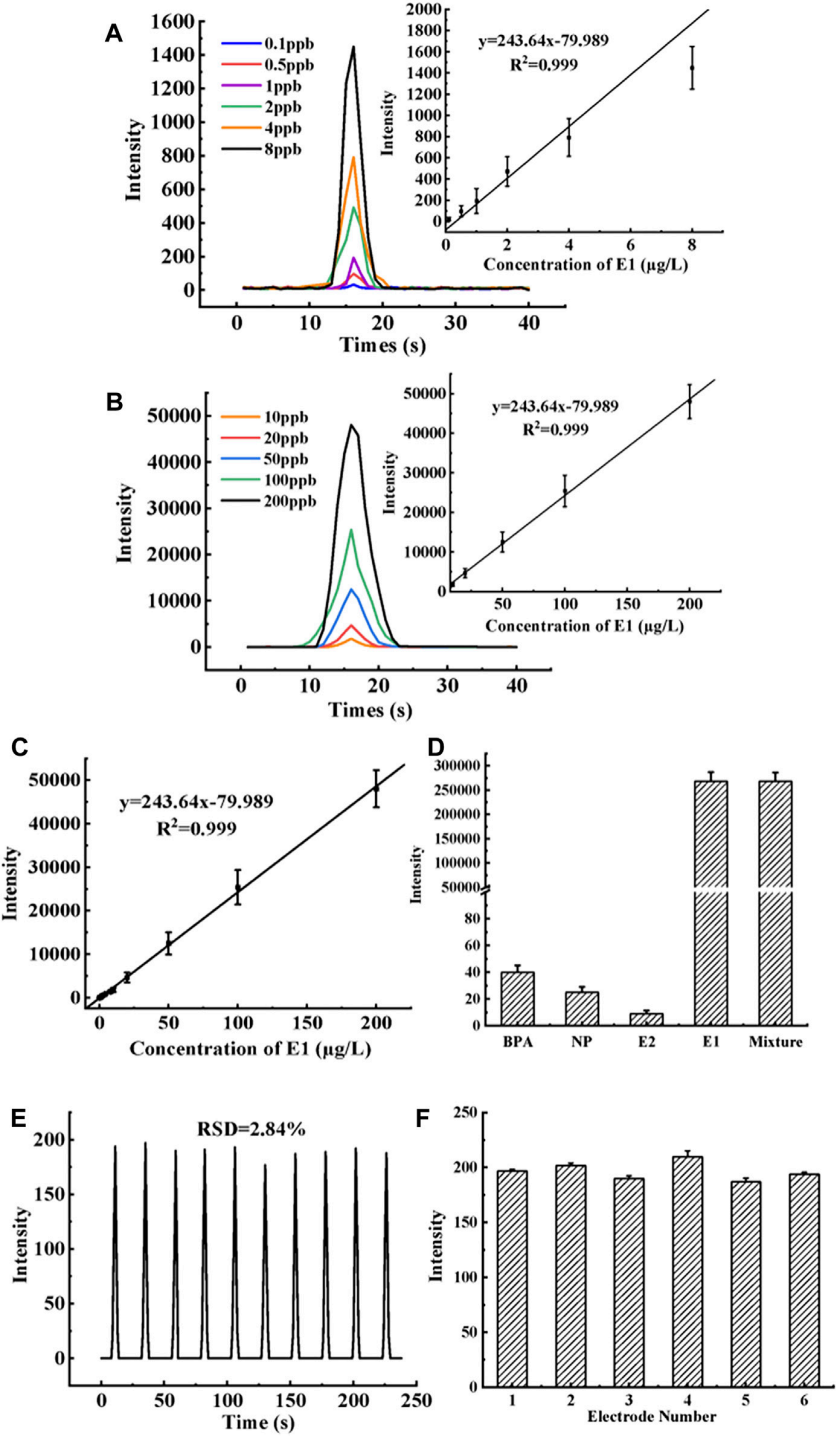
**(A)** The curve graph of ECL response intensity with time. The six curves in the figure represent E1 standard solutions of different concentrations ranging from 0.1 μg/L to 8 μg/L. The illustration in **(A)** is the linear calibration curve of ECL response intensity when E1 concentration is the corresponding curve concentration in this range. **(B)** Same as **(A)**, except that the concentration interval of E1 is from 10 μg/L to 200 μg/L. **(C)** Same as **(A)**, except that the concentration interval of E1 is from 0.1 μg/L to 200 μg/L. **(D)** MIP-ECL sensor is used to detect E1 interfering substances such as 1 mg/L E2, 1 mg/L BPA, and 1 mg/L NP respectively. **(E)** The curve of ECL changes over time during 10 consecutive detections by the MIP-ECL sensor is used to evaluate the stability of the sensor. **(F)** Reproducibility of six parallel electrodes labeled 1 to 6.

After the electrodes of the MIP-ECL sensor were modified in layers according to Section 2.5, they were immersed in an E1 solution of different concentrations and continuously stirred for 8 min. Then sensors were removed and rinsed off the non-specific bound E1. In measurement, they were placed in a PBS/TPA buffers solution (without E1) to test the ECL signal response. The recorded ECL curves showed that when the concentration of E1 in the solution increased, the ECL response signal also increased. Furthermore, the E1 concentration and ECL intensity were fitted by linear regression, and the linear range was determined to be an E1 concentration between 0.1 μg/L to 200 μg/L. As shown in Figures 7A–C, the linear regression equation is ΔY = 243.64x-79.989, where the dependent variable (ΔY) is the difference between the ECL intensity of the sample solution and that of the blank solution (without E1). The independent variable (X) is the concentration of E1 in the sample solution. The linear correlation coefficient (*R* ^2^) of the equation is 0.999, and the detection limit of this method is 0.0047 μg/L. Since the linear range of the sensor is wide, in order to show the luminescence intensity of E1 at each concentration more clearly, we divided the concentration of E1 from 0.1 μg/L to 200 μg/L into two sections and drew the diagram respectively. Figure 7A shows the curve graph of ECL response intensity with time. The six curves in the figure represent E1 standard solutions of different concentrations ranging from 0.1 μg/L to 8 μg/L. In the figure, with the increase of E1 concentration, ECL intensity also increases gradually. The illustration in Figure 7A shows the linear regression equation of the corresponding E1 concentration and peak value of ECL intensity. Five parallel experiments were conducted at each concentration level. Similar to Figure 7A, the 5 curves in Figure 7B respectively represent the concentration of E1 standard solution ranging from 10 μg/L to 200 μg/L. The illustration in Figure 7B is the linear regression equation of ECL response intensity when E1 concentration is the corresponding curve concentration in this range. Figure 7C shows the linear regression equation of all E1 concentrations (i.e., from 0.1 μg/L to 200 μg/L) and the ECL response intensity. In Figures 7A–C, each concentration level is the average of five parallel results. As seen from Table 3, the LOD of the new method is 10 times lower than that of the LC-MS/MS method, which also indicates that the new method is more sensitive at detecting the target compound E1 in the sample. The quantitative parameters of the new MIP-ECL sensor method and the classical LC/MS-MS method are listed in Table 3, including the linear regression equation, linear interval, *R* ^2^, and LOD.

**TABLE 3:** List of quantitative parameters of new and classical methods for E1 detection.

Means	MIP-ECL sensor	LC-MS/MS
Linear interval (μg/L)	0.1–200	1–200
Linear regression equation	Y = 243.64x-79.989	Y = −1782.7 + 4070.2x
*R* ^2^	0.999	0.9991
LOD (µg/L)	0.0047	0.052

### 3.5 Performance evaluation of new sensor

To begin with, it is essential to assess a sensor’s selectivity towards the target molecule to gauge its overall efficacy. In this study, we conducted a comprehensive analysis of the novel MIP-ECL sensor’s specificity towards E1 molecules. Specifically, we utilized the MIP-ECL sensor to detect E1 interfering substances such as 1 mg/L E2, 1 mg/L BPA, and 1 mg/L NP, and recorded the maximum ECL response intensity value (as illustrated in Figure 7D). Our findings reveal that the maximum ECL response intensity of these interfering substances was remarkably low, underscoring the superior selectivity of the new sensor towards E1 molecules, thus making it an ideal tool for targeted E1 detection.

In addition, the stability of the sensor is another important factor in terms of performance evaluation. In this study, the MIP-ECL sensor was used to detect the E1 standard solution of 1 μg/L for 10 consecutive cycles, and the relative standard deviation (RSD) of the ECL response intensity obtained was counted to evaluate the stability of the novel sensor. Figure 7E shows the curve diagram of ECL intensity changes over time during 10 consecutive detections by the MIP-ECL sensor. The figure shows that the RSD of 10 response intensities of the new sensor is about 2.8%, indicating that the sensor has a good stability. Besides, in order to prove that the sensor performance is not affected by aging, the prepared sensor was set aside for a week and then used to compare with the E1 intensity of the newly prepared sensor. It was found that the ECL intensity of the two sensors did not change significantly.

Finally, the reproducibility of the sensor is important for its performance as well. In this study, the electrode of the new MIP-ECL sensor was cleaned and polished to remove all coatings, then the electrode was modified again, and the process was repeated five times to evaluate the reproducibility of the new sensor. Reuse of the sensor can not only save resources but also facilitate the change of specific target molecules, which can be flexibly used in the actual determination process. The specific research process is to carry out a reproducibility experiment on 6 sensor electrodes in parallel according to the above steps. Each electrode is repeatedly polished and recoated 5 times, and its ECL response intensity is tested under the E1 concentration of 1 μg/L and optimal conditions after each completed recoating of the electrode. The average and RSD values of ECL response intensity for the five trials were calculated to evaluate the reproducibility of the MIP-ECL sensor. In Figure 7F, the *X*-axis indicates that the six parallel electrodes are marked with numbers 1 to 6 respectively. The *Y*-axis represents the average value of the maximum ECL response intensity when 1 μg/L E1 is detected with the same electrode that has been polished and recoated 1 to 5 times. It is clearly evident that the ECL signals of the six electrodes are nearly the same. The average values of the maximum ECL response intensity of the five tests on electrodes 1 to 6 are between 187 and 210 and the RSD values of the maximum ECL response intensity of the five tests on electrodes 1 to 6 lie between 1.44% and 5.39%. These results demonstrate the ease of reconstruction of the molecularly imprinted coating without affecting the ECL’s signal strength or sensitivity, demonstrating its excellent reproducibility.

### 3.6 Real sample testing

One liter of water samples was taken from three rivers (A, B, and C) in Fuzhou city. A was a branch river near the old city market; B was a branch river near the new city business district; and C was a main river in the suburbs. A bunch of 3 mL samples was taken from sampling points A, B, and C; and after being filtered through a 0.45 μm filter and getting pH adjusted into the electrolytic cell, the samples were placed in the photomultiplier tube for ECL detection. The obtained ECL response intensity is taken as the Y value in the formula ΔY = 243.64x-79.989, and the calculated X value is the E1 concentration in the sample. The solutions of actual samples A, B, and C were measured and calculated according to the above steps, and the content of E1 was 2.8620, 1.7143, and 0.7251 μg/L respectively. In order to verify the accuracy of quantitative results by using the new sensor method, the same samples A, B, and C solutions were re-quantified using the traditional LC-MS/MS method. The quantitative results of the two analysis methods are nearly identical, as shown in Table 4, which further indicates that the quantitative analysis of E1 in actual samples by using the new sensor method is accurate and reliable.

**TABLE 4:** In the actual water sample, quantitative results of E1 were compared after analysis by MIP-ECL sensor and LC-MS/MS method.

Sample	Quantitative results (μg/L)		Relative deviation (%)
	MIP-ECL sensor	LC-MS/MS	
A	2.8620 ± 2.70	3.11 ± 4.63	4.1
B	1.7143 ± 3.45	1.69 ± 5.27	0.7
C	0.7251 ± 5.39	0.723 ± 5.98	0.1

As Table 4 highlights, the concentration of E1 in sampling point C is lower than sampling point A and B. This is because the suburban trunk rivers have larger volumes of water and larger flow momentums, which can dilute the concentration of pollutants. The concentration of E1 in sampling point A is about twice as high as that in sampling point B due to the pollutants such as livestock excreta entering the river near the market. By comparing the quantitative results of the same sample detected by the two methods, it is found that the relative deviation of the quantitative results is less than 4.1%, which indicates that the quantitative accuracy of the new sensor method developed in this paper is comparable to that of the standard LC-MS/MS. In summary, the new E1 sensor has excellent performance and can meet the requirements of routine quantitative detection of E1 in various types of samples in the laboratory.

## 4 Conclusion

In this work, we have successfully developed a novel MIP-ECL sensor that is sensitive and specific for accurate quantitative toward E1 along with an additional benefit of rapid reconstruction. To begin with, the ECL material Ru-bpy was immobilized on the gold electrode through electrostatic adsorption of MWCNTs and ion exchange of Nafion to form a solid-state light-emitting electrode. Then, the MIP-ECL sensor was conceived by immobilizing a MIP with specific selectivity onto the electrode. The electrochemical properties of this MIP-ECL sensor and the electrochemical luminescence properties of the E1 molecule in solution were investigated, indicating that E1 has an electro-catalytic effect on the reduction current of Ru-bpy as well as a strong luminescence signal to improve the sensitivity of MIP-ECL sensors. At the same time, co-reagent, solution medium, and pH parameters were optimized. TPA as a co-reagent can greatly improve the luminescence intensity of MIP-ECL sensors, and the luminescence signal is strongest when the pH of PBS is 7.4. In terms of the selectivity of MIP-ECL sensors, they show relatively low ECL signal to three E1 interfering substances (E2, BPA, and NP), which proves its specificity to the E1 molecule and can be used to detect the concentration of E1 in various sample solutions. When the E1 concentration range is from 0.1 μg/L to 200 μg/L, it can obtain a good linear fitting with the ECL signal intensity. The quantitative equation of the sensor is Δy = 243.64x-79.989; *R* ^2^ is 0.999, and LOD is 0.0047 μg/L. The MIP-ECL sensor is fast, highly sensitive, easy to operate, and suitable for field analysis. Moreover, the quantitative results are accurate and can be used for routine quantitative analysis of E1 in the laboratory. Certainly, the new MIP-ECL sensor developed in this study also has some drawbacks or shortcomings. Firstly, in the preparation of electrode sensitive films, it is necessary to optimize parameters further, such as the thickness of the MIP functional film and the elution efficiency of template molecules, to ensure the imprinting capacity. Secondly, the sensitivity of the sensor is acceptable but not the best, compared with other studies listed in Table 1. Our future work is to attempt a new immobilization method for the luminescent reagent Ru-bpy, and to construct a multiple signal amplification system using MIP and other nanomaterials to enhance the sensitivity of the sensor.

## Data Availability

The original contributions presented in the study are included in the article/Supplementary Material, further inquiries can be directed to the corresponding author.
