# Supplementary material for: Stable and reproducible MIP-ECL sensors for ultra-sensitive and accurate quantitative detection of Estrone
Source: Front Bioeng Biotechnol. 2024 Feb 9;12:1329129. doi: 10.3389/fbioe.2024.1329129 (PMC10893587; doi:10.3389/fbioe.2024.1329129)
Supplement: Supplementary file 1 [file DataSheet1.doc]

Supplementary Material

# Stable and Reproducible MIP-ECL Sensors for Ultra-Sensitive and Accurate Quantitative Detection of Estrone

Jie Cao*, Xiao-Ying Chen

*** Corresponding Author:** E-mail address: [caojie@fjpsc.edu.cn](mailto:caojie@fjpsc.edu.cn), Tel.: +86 13489963244

# Electrochemical properties of the modified electrodes

The electrochemical properties of MWCNT/Nafion/Au electrodes were studied by cyclic voltammetry (CV). FIG. S1-A shows the CV changes obtained by the detection of 1 mmol/L Ru-bpy in PBS solution with different modified electrodes. The black line a is an unmodified gold electrode, and the red line b is the MWCNT/Nafion/Au electrode. On the unmodified electrode, there is a small reduction peak current at about 0.7v. On the MWCNT/Nafion/Au electrode, since the MWCNT/Nafion membrane could enrich and adsorb Ru-bpy, the peak current increased significantly, indicating that the Nafion could effectively adsorb Ru-bpy. Meanwhile, as MWCNT has high surface atomic activity, which can promote the rapid diffusion of Ru-bpy in MWCNT/Nafion membrane, MWCNT also increases the electrode surface area, has good conductivity, and increases the adsorption capacity of Ru-bpy, thus increasing the current.

In order to prove that Ru-bpy has good CV characteristics in both liquid and solid phases, the Ru-bpy/MWCNT/Nafion/Au electrode was scanned in PBS solution. At the same time, MWCNT/Nafion/Au electrode was used to detect the CV of Ru-bpy solution. The results are shown in Figure S1-B. It is found that the CV current curves of Ru-bpy in the liquid phase and the solid phase are similar, and there is an obvious reduction peak current at ~0.7V, indicating that Ru-bpy fixed on the electrode maintains good electrochemical activity.

The effect of Ru-bpy/MWCNT/Nafion/Au electrode on the presence or absence of E1 molecules in the solution was investigated by electrochemical scanning. According to the results of FIG. SI-C, the reduction peak potential of Ru-bpy has little change regardless of the presence of E1 molecules in the solution or not, indicating that the electrochemical activity of Ru-bpy in the modified film is not affected by E1 molecules. Compared with the CV curve of the solution without E1 molecules, the reduction peak current of Ru-bpy increases when E1 is added into the solution, which indicates that E1 has an electrocatalytic effect on the reduction current of Ru-bpy. This phenomenon was also mentioned in Alexey V. Kuzikov's paper, which also proves the correctness of this result [1].

**Reference**

1. Alexey V. Kuzikov, Rami A. Masamrekh, Tatiana A. Filippova, Yaraslau I. Haurychenka, Andrei A. Gilep, Tatsiana V. Shkel, Natallia V. Strushkevich, Sergey A. Usanov, Victoria V. Shumyantseva, Electrochemical oxidation of estrogens as a method for CYP19A1 (aromatase) electrocatalytic activity determination, Electrochimica Acta, 2020, 135539. https://doi.org/10.1016/j.electacta.2019.135539.

**A**

**C**

**B**

**Figure S1:** Electrochemical properties of the modified electrodes. (A) The CV diagrams of the unmodified electrode (a) and the MWCNT/Nafion modified electrode (b) in PBS solution containing Ru-bpy. (B) The CV diagrams of Ru-bpy in aqueous (black, MWCNT/nafion/Au electrode) and solid (red, Ru-bpy/MWCNT/Nafion/Au electrode in PBS) phases. (C) The CV diagrams of 1 mg/L E1 (a) and PBS solution (b) at Ru-bpy/MWCNT/Nafion modified electrode.
